# Auditory spatial representations of the world are compressed in blind humans

**DOI:** 10.1007/s00221-016-4823-1

**Published:** 2016-11-11

**Authors:** Andrew J. Kolarik, Shahina Pardhan, Silvia Cirstea, Brian C. J. Moore

**Affiliations:** 1 Department of Psychology, University of Cambridge, Downing Street, Cambridge, CB2 3EB UK; 2 Vision and Eye Research Unit (VERU), Postgraduate Medical Institute, Anglia Ruskin University, YST 215, Young Street, Cambridge, CB1 1PT UK; 3 Centre for the Study of the Senses, Institute of Philosophy, University of London, Senate House, Malet Street, London, WC1E 7HU UK

**Keywords:** Blindness, Spatial hearing, Auditory distance, Multisensory plasticity, Sound localization

## Abstract

Compared to sighted listeners, blind listeners often display enhanced auditory spatial abilities such as localization in azimuth. However, less is known about whether blind humans can accurately judge distance in extrapersonal space using auditory cues alone. Using virtualization techniques, we show that auditory spatial representations of the world beyond the peripersonal space of blind listeners are compressed compared to those for normally sighted controls. Blind participants overestimated the distance to nearby sources and underestimated the distance to remote sound sources, in both reverberant and anechoic environments, and for speech, music, and noise signals. Functions relating judged and actual virtual distance were well fitted by compressive power functions, indicating that the absence of visual information regarding the distance of sound sources may prevent accurate calibration of the distance information provided by auditory signals.

## Introduction

The ability to produce, retain, and update an accurate internal representation of the external world in the absence of a primary sensory modality is an important topic in psychology and neuroscience, and a large number of studies have attempted to address this by investigating the effects of visual loss on the ability to perform spatial tasks using auditory cues (for reviews, see Collignon et al. 2009; Voss et al. 2010). Accurate spatial representations based on sound are particularly important for blind people, as they underlie successful navigation performance (for a review, see Schinazi et al. 2016). It is clear that blind listeners do have a sense of auditory space. However, how this space is calibrated and how the calibration is maintained are not clear. The sense of auditory space for sounds within grasping distance may be calibrated via haptic feedback. For more distant sounds, it is possible that audiomotor feedback (the use of systematic changes in auditory stimuli resulting from self-motion) can be used to provide accurate calibration of auditory space in the absence of vision (Jones 1975; Ashmead et al. 1989; Lewald 2013). For example, rotation of the head about its vertical axis leads to corresponding changes in interaural time delay (ITD) and interaural level difference (ILD) cues for azimuthal localization, and the correspondence between the two might serve to calibrate the ITD and ILD cues (O’Regan and Noë 2001; Lewald 2002a).

The perceptual deficiency hypothesis posits that without vision to aid in calibrating audition, auditory spatial abilities may be poorer for those with visual loss than for sighted individuals (Axelrod 1959; Jones 1975). An opposing viewpoint is that blind individuals would have enhanced spatial abilities using sound due to extensive experience in extracting information from sound and reliance on sound (Rice 1970) and because compensatory processes, such as cortical reorganization, may enhance auditory spatial performance in certain conditions (Voss and Zatorre 2012). Both viewpoints have received support in the literature (for a review, see Voss et al. 2010). Some studies, mainly focusing on the ability to localize in azimuth or to detect changes in the azimuth or distance of sounds, have shown that blind listeners have sound localization abilities similar to or better than those for normally sighted listeners (Lessard et al. 1998; Doucet et al. 2005). However, blind individuals performed more poorly than sighted controls in an auditory spatial bisection task in which subjects reported whether the second sound of three sounds was closer to the left (first) or right (third) sound (Gori et al. 2014; Vercillo et al. 2016). Vertical sound localization has been reported to be less accurate for blind than for sighted listeners in both quiet conditions (Lewald 2002b) and in the presence of background noise (Zwiers et al. 2001). Evidence from Voss et al. (2015) suggests that a trade-off may occur between the horizontal and vertical planes for blind participants localizing sounds monaurally, such that learning to utilize monaural spatial cues for horizontal localization may come at the cost of using the monaural cues to localize sounds in terms of elevation.

The conditions under which visual loss leads to enhancement or worsening in auditory spatial perception have not been fully established. In particular, little is known about how visual loss affects the perception of distance using auditory cues. The aim of the current study was to compare the fidelity of the spatial representation of distance in extrapersonal space (farther than 1 m from the listener) for blind and normally sighted listeners using virtual auditory cues alone. The two main distance cues for stationary sounds in extrapersonal space are sound level (Coleman 1963; Mershon and King 1975) and direct-to-reverberant energy ratio, or D/R (Mershon and King 1975; Zahorik 2002a). Level generally provides more accurate information about distance [see Kolarik et al. (2016a) and Zahorik et al. (2005) for reviews]. However, if the room reverberation time is sufficiently long, the two cues can be equally effective (Kolarik et al. 2013a). Blind listeners usually show supra-normal performance for relative auditory distance judgments (Ashmead et al. 1998; Voss et al. 2004; Kolarik et al. 2013b), but a deficit in relative auditory distance judgments by both early-blind children and adults was reported by one study (Cappagli et al. 2015).

Absolute judgments of distance to single, static sources necessitate the use of a topographic representation of the auditory world without any reference or comparison sound sources. For normally sighted listeners, the visual system provides more accurate distance information than the auditory system (Da Silva 1985; Loomis et al. 1998), and visual information would normally be used to fine-tune neural representations of distance and to calibrate auditory information about distance. Lack of visual information, therefore, may lead to lower accuracy of absolute distance judgments by blind listeners if audiomotor feedback is not sufficient to calibrate auditory distance, consistent with the perceptual deficiency hypothesis. This viewpoint has been supported by studies showing that absolute auditory distance perception was less accurate for early-onset blind than for sighted participants, both for 800-Hz tones (Wanet and Veraart 1985) and for white noises (Macé et al. 2012). It is currently unclear whether room reverberation affects absolute distance judgments for the blind, as the room reverberation time was not reported for these studies. Also, judgments were limited to sound sources in peripersonal space (sounds within reaching and grasping distance, up to approximately 1 m away from the individual). Another study showed that blind participants were less accurate than sighted participants at judging the distance of speech sounds simulated to be in extrapersonal space (Kolarik et al. 2013c). However, this experiment was conducted using a virtual anechoic room only, and room reverberation was not investigated. There is currently a gap in knowledge regarding the effect of visual loss on absolute distance judgments in extrapersonal space for different acoustic environments and for stimuli other than speech.

The current study used virtualization methods to investigate whether absolute distance judgments for virtual sounds in extrapersonal space would be less accurate for early-blind participants than for sighted controls, consistent with the perceptual deficiency hypothesis, and whether this generalized across anechoic and reverberant virtual rooms, and for a range of stimuli (speech, music, and noise, which differ in their spectro-temporal characteristics). The virtualization methods used in the current study allowed control over stimulus parameters including room reverberation time. To our knowledge, this is the first time that absolute distance judgments for blind and sighted individuals in both virtual anechoic and reverberant rooms have been assessed.

## Materials and methods

### Participants

There were two groups of participants: early-onset blind (defined here as having lost their sight between birth and 5 years of age, *n* = 10, 4 males and 6 females, mean age 45 years, range 25–69 years; see Table 1 for details) and normally sighted (*n* = 11, 5 males and 6 females, mean age 41 years, range 20–67 years). The blind participants were either totally blind or had some light perception only, and fell into categories 4–5 of the World Health Organization classification (World Health Organization 1989). Sighted participants reported normal or corrected-to-normal vision. All participants had normal or near-normal hearing, defined as better-ear average (BEA) hearing threshold across the frequencies 500, 1000, 2000, and 4000 Hz equal to or less than 25 dB HL, as measured using an AS608 Interacoustics audiometer following the procedure recommended by the British Society of Audiology (2011). Participants were paid for taking part. The experiments followed the tenets of the Declaration of Helsinki. Informed consent was obtained from all participants following an explanation of the nature and possible consequences of the study. The experiments were approved by the Anglia Ruskin University Ethics Panel.

**Table 1:** Details of blind participants

	Sex, age, age of onset of vision loss (years)	Cause of vision loss	Visual status, WHO category
B1	M, 46, 5	Stickler’s syndrome, retinal detachment	No light perception, 5
B2	F, 52, 5	Macular degeneration	Light perception, 4
B3	M, 62, birth	Retinopathy of prematurity	No light perception, 5
B4	M, 25, 3	Retinoblastoma	No light perception, 5
B5	M, 38, birth	Familial exudative vitreoretinopathy	No light perception, 5
B6	F, 52, 1.5	Glaucoma	No light perception, 5
B7	F, 26, 2	Norrie disease	Light perception, 4
B8	F, 42, 1	Retinoblastoma	No light perception, 5
B9	F, 69, 3	Glaucoma	Light perception, 4
B10	F, 42, birth	Retinopathy of prematurity	No light perception, 5

### Apparatus and stimuli

**Fig. 1:**
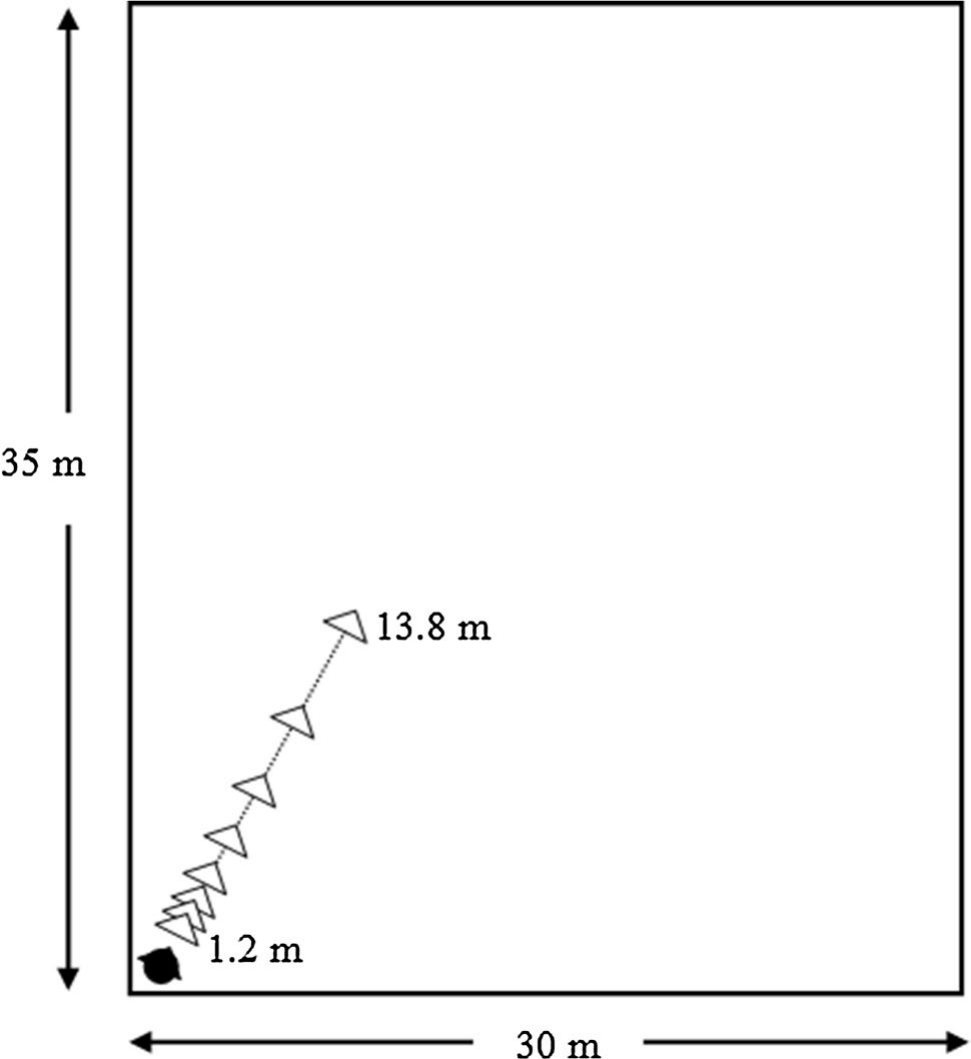
Schematic of the virtual room and the configuration of the participant and sound sources. The participant’s position is shown by the *black symbol*, and the simulated sound source positions are shown by *open triangles*

Signals were generated by an ESI UGM96 sound card, using a custom-written MATLAB script (Mathworks, Inc.) with a response interface on a Lenovo T420 ThinkPad laptop. Following previous studies that tested the auditory abilities of blind participants (Teng et al. 2012; Rowan et al. 2013), blind participants were tested in a quiet laboratory room or in a quiet room in their homes if they preferred. Four blind and four sighted controls were tested at their homes. Stimuli were presented via closed-back Sennheiser HDA200 headphones, which have a transducer mounted within a hard shell casing, providing about 30 dB of passive attenuation of ambient noise, to ensure that extraneous background noises were inaudible. Digital filtering was used to correct the frequency response of the headphones so as to simulate free-field presentation. The stimuli were generated using methods described in previous studies (Kolarik et al. 2013a, b, c, d). A large virtual room measuring 30 × 35 × 10 m was simulated using an image source model, or ISM (Lehmann and Johansson 2008). The virtual room was either anechoic, so that level cues only were available, or reverberant, so that level and D/R cues were available. The reverberation time (*T*_60_, the time taken for the sound level to fall by 60 dB) was 700 ms, comparable to that used in a previous study (Zahorik 2002b). The ISM synthesizes a room impulse response (RIR) between a virtual source and receiver separated by a specified distance. Convolution of the RIR with a sound sample provides a virtual sample of the sound heard within the simulated room, at the specified virtual distance. The simulated position of the participant was in the near-left corner 1 m from each wall at 1 m height, facing forward into the room at 30° relative to the long wall (Fig. 1). Stimuli were presented at 1 m height, at 0° azimuth relative to the front of the head and at 0° elevation.

The stimuli consisted of speech, music, and broadband noise. One speech stimulus was a sentence spoken at a conversational level by a male, randomly selected from the Bench–Kowal–Bamford corpus (Bench et al. 1979). The sentence was sampled at 22.05 kHz and had a duration of 1.5 s, chosen to match stimuli previously used in a distance discrimination study with sighted participants (Akeroyd et al. 2007). Music stimuli consisted of a 7.3-s segment of a jazz trio (piano, bass and drums) sampled at 22.05 kHz, previously used to investigate the influence of upper cutoff frequency on preferences for music using hearing aids (Moore et al. 2011). Noise stimuli were broadband (0.6–12 kHz) white noise bursts with 90-ms duration and 10-ms rise/fall time, sampled at 44.1 kHz, chosen to match stimuli previously used to investigate auditory distance discrimination by sighted and blind participants (Voss et al. 2004; Kolarik et al. 2013b).

The distances of the simulated sound sources were defined as spatial coordinates located in extrapersonal space (here defined as farther than 1 m) located directly in front of the simulated participant position at 1.22, 1.72, 2.44, 3.45, 4.88, 6.90, 9.75, and 13.79 m, based on distances used in a previous distance estimation study by Zahorik (2002a). Stimuli were spatially rendered by convolving a nonindividualized head-related transfer function (HRTF) with the direct sound component. The HRTF was obtained from publicly available HRTF measurements made by Gardner and Martin (1995) using a Knowles Electronics Manikin for Acoustics Research under anechoic conditions for a distance of 1.4 m between the source and the manikin. Previous studies investigating spatial localization ability (Voss et al. 2011) and distance discrimination (Kolarik et al. 2013b) for blind participants also used this set of HRTF measurements. Otani et al. (2009) showed that HRTFs are approximately independent of source distance for distances greater than 1 m. HRTFs measured at a fixed distance of 1 m have been used previously to study absolute distance judgments by sighted participants (Brungart and Scott 2001). Stimuli were processed offline before being saved in computer files for access during the experiment. The mean presentation level of the stimuli was 66 dB SPL (unweighted) for a virtual distance of 1 m from the participant’s position. The level decreased with increasing virtual distance. For a discussion of the limitations of the methods used for simulation, see Kolarik et al. (2013b).

### Procedures

Participants were asked to imagine themselves to be seated in a rectangular room of an unspecified size, listening to sounds emitted from a loudspeaker situated at various distances in front of them. The participants were informed that the stimuli would be simulated and that they should use only acoustic information regarding their perception of the environment. Sighted participants and blind participants with light perception were instructed to keep their eyes closed during the experiment, following Vercillo et al. (2015).^1^ They were monitored by the experimenter throughout the experiment to ensure that this instruction was followed. Single stimuli were presented in random order at the virtual distances listed above. Participants verbally reported the apparent distance of each sound source in meters and centimeters, or feet and inches if they preferred. Responses were recorded by the experimenter using the response interface. Participants were instructed to report zero meters or feet if they perceived the sound to originate within the head. No training was provided. No limitation was imposed on response time, and no feedback was given.

Within a single block of trials, stimulus type (speech, music or noise) and condition (anechoic or reverberant room) were kept constant. There were 10 repetitions per virtual distance, and thus 80 trials in each block. Measurements for the 3 stimulus types and 2 conditions were obtained within a single session (6 blocks = 480 trials in total). The order of presentation of the 6 blocks was randomized. The experiment lasted approximately 1 h and 40 min.

## Results

### Auditory distance judgments

**Fig. 2:**
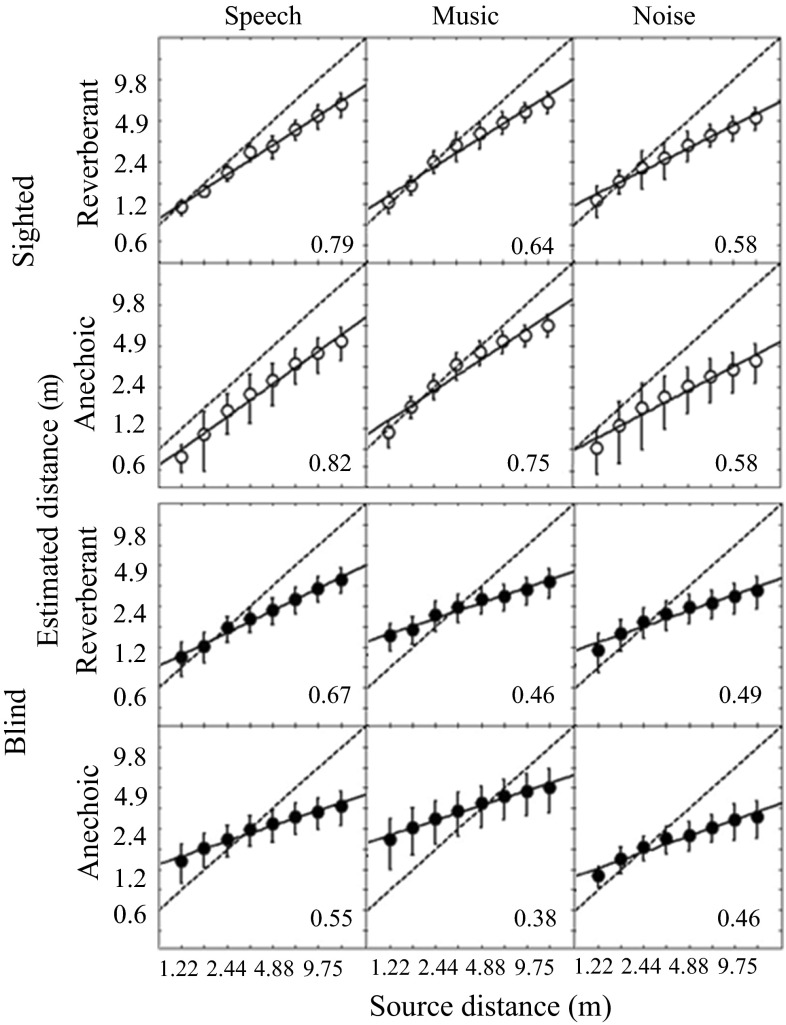
Auditory distance judgments as a function of virtual source distance. *Symbols* show geometric mean data for sighted participants (*upper six panels*, *open circles*) and blind participants (*lower six panels*, *filled circles*). Results are shown in separate panels for speech, music, and noise stimuli. The *upper* and *lower panels* for each group show results for the simulated reverberant and anechoic rooms, respectively. *Error bars* indicate ±1 standard error across participants. Linear fits to the data on log–log coordinates are shown by *solid lines*; the slope is reported in the bottom right corner of each panel (equivalent to the *a* parameter of the compressive power function proposed by Zahorik et al. 2005). *Dashed lines* indicate where the points would lie if performance was perfect

No responses of zero distance to the sound source were reported by any of the participants, indicating that all sounds were externalized. The accuracy of participants’ distance judgments is shown in Fig. 2, which shows apparent distance judgments plotted as a function of virtual distance on log–log coordinates. Open and filled symbols show geometric mean results for sighted and blind participants, respectively. Geometric means were used following Zahorik (2002a). The upper and lower panels show results for simulated reverberant and anechoic rooms, respectively. For each group (sighted or blind), results are shown separately for speech, music, and noise stimuli.

Zahorik et al. (2005) showed that compressive power functions of the form* r*′ = *kr* ^*a*^ gave good fits to normally sighted participants’ judgments of distance using auditory cues, where* r*′ is the estimate of perceived distance, *r* is the actual source distance, and *k* and *a* are adjustable parameters. Slopes of linear fits to the current data on logarithmic coordinates are equivalent to the *a* parameter and are reported in the bottom right of each panel of Fig. 2.

For the normally sighted participants, the estimated distance was close to the actual distance for small distances but the distance was underestimated for greater distances, and the amount of underestimation increased as virtual distance increased. The slopes for speech stimuli were significantly steeper than those for noise stimuli [anechoic: *t*(12) = 4.54, *p* < 0.001, reverberant: *t*(12) = 5.24, *p* < 0.001], indicating more veridical distance judgments for speech sounds in both anechoic and reverberant simulated environments. The slope for music stimuli fell between those for the speech and noise stimuli, in both the anechoic and reverberant virtual rooms. For the blind participants, the distances to nearby sources were overestimated while the distances to farther sources were underestimated to a greater extent than for the sighted participants. Thus, the range over which distances were judged to change was markedly compressed relative to the actual range of distances. In the reverberant condition, the slope was significantly steeper for speech stimuli than for music [*t*(12) = 4.42, *p* < 0.001] and noise [*t*(12) = 3.41, *p* < 0.01] stimuli. Slopes for music and noise were similar in the reverberant condition. In the anechoic condition, the slope was steeper for speech stimuli than for music [*t*(12) = 2.63, *p* < 0.05] but not noise [*t*(12) = 1.39, *p* < 0.01]. The slope was significantly less for the blind than for the sighted group in all but one of the conditions: speech [anechoic: *t*(12) = 3.63, *p* < 0.01; reverberant: *t*(12) = 2.82, *p* < 0.05], music [anechoic: *t*(12) = 5.24, *p* < 0.001, reverberant: *t*(12) = 3.89, *p* < 0.001], and noise [anechoic: *t*(12) = 2.67, *p* < 0.05]. For noise in a reverberant virtual room, there was a trend in the same direction, but it was not significant [*t*(12) = 1.85, ns]. A mixed-model ANOVA of the slopes with reverberation time and stimulus as within-subjects factors and blindness as a between-subjects factor showed main effects of stimulus [*F*(2, 38) = 6.60, *p* < 0.01] and blindness [*F*(1, 19) = 4.41, *p* < 0.05]. No other main effects or interactions were significant (all *p* > 0.05). In summary, these findings indicate that blind participants’ judgments of distance are compressed relative to those for sighted participants.

**Fig. 3:**
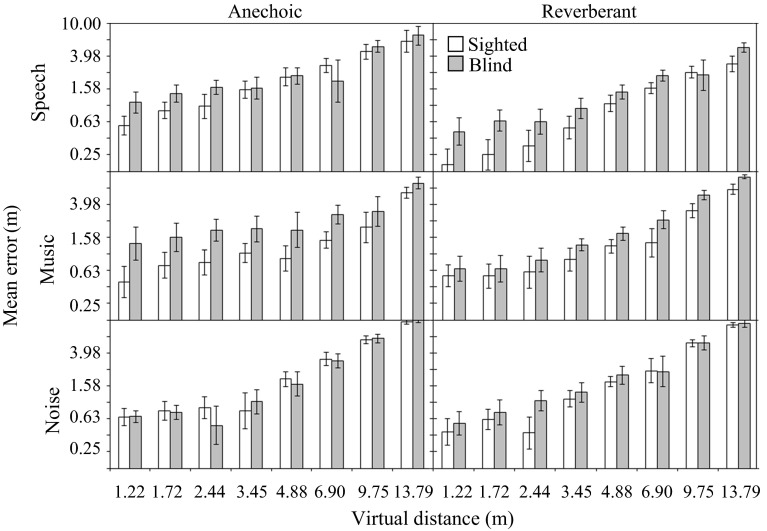
Mean absolute errors of the distance judgments for sighted (*open bars*) and blind (*gray bars*) participants. The *top*, *middle,* and *bottom panels* show results for speech, music, and noise, respectively. The *left* and *right panels* show results for the simulated anechoic and reverberant rooms, respectively. Both axes are logarithmic. *Error bars* indicate ±1 standard error across participants

To examine the precision of distance judgments, the mean unsigned error (the difference between judged and virtual distance for each trial regardless of direction) was calculated. Figure 3 shows the mean errors for sighted and blind participants for each condition. The top, middle, and bottom panels show errors for speech, music, and noise, respectively. The left and right panels show errors for anechoic and reverberant rooms, respectively. For both groups, errors increased with increasing virtual distance, and judgments made in the reverberant room tended to be more accurate. Sighted participants tended to be more accurate than blind participants, especially for closer sound sources, and for speech in a reverberant virtual room and for music in an anechoic virtual room. A mixed-model ANOVA on the error scores with distance, reverberation time, and stimulus as within-subjects factors and blindness as a between-subjects factor showed main effects of distance [*F*(7, 133) = 67.16, *p* < 0.001], reverberation time [*F*(1, 19) = 8.51, *p* < 0.01], and blindness [*F*(1, 19) = 5.56, *p* < 0.05], and significant interactions between stimulus and reverberation time [*F*(2, 38) = 3.72, *p* < 0.05], and distance and reverberation time [*F*(7, 133) = 3.03, *p* < 0.01]. No other main effects or interactions were significant (all *p* > 0.05). A posteriori tests with Bonferroni correction showed that error scores were significantly larger in the anechoic room than in the reverberant room for the speech stimulus only. In summary, the results show that errors were significantly higher for the blind than for the sighted participants.

The inherent variability of the judgments was assessed using the standard deviation (SD) of the ten judgments for each participant and each condition. A mixed-model ANOVA of the SDs, with distance, reverberation time, and stimulus as within-subjects factors and blindness as a between-subjects factor, showed main effects of stimulus [*F*(2, 38) = 4.51, *p* < 0.05] and distance [*F*(7, 133) = 7.08, *p* < 0.001], and interactions between blindness and distance [*F*(7, 133) = 3.63, *p* < 0.001], and stimulus and distance [*F*(14, 266) = 1.91, *p* < 0.05], but no main effect of blindness [*F*(1, 19) = 4.23, ns]. Although there was a trend for sighted participants to give higher judgment variability than blind participants at greater distances, a posteriori tests with Bonferroni correction showed no significant differences between the groups. Thus, the inherent variability of the distance judgments was not different for the blind and sighted participants.

### Subsidiary experiment: nonvisual, nonauditory distance judgments

It is possible that the differences across groups described above were due to an underlying difference between blind and sighted participants in their ability to estimate distances in general. To assess this, we obtained distance judgments for a nonauditory and nonvisual task in which participants were instructed to walk forwards over distances of 2, 5, and 10 m. Previous work has shown that for a visual distance task, both walked and verbal responses are controlled by the same internal variable of perceived distance and that walking responses are not regulated by another internal variable governed by the number of paces or walking time to reach objects (Philbeck and Loomis 1997). Assuming that this also applies to the nonvisual and nonauditory walking task in our subsidiary experiment, the results should give an indication of the general ability to judge perceived distance that can be compared to the perceived auditory distance judgments given by participants in the main experiments.

Groups consisted of blind (*n* = 9, including 6 participants who took part in the main experiment, 2 males and 7 females, mean age 44 years, range 22–69 years), and normally sighted participants (*n* = 9, 5 males and 4 females, mean age 39 years, range 22–67 years). All participants had normal hearing, and blind participants had total visual loss or light perception only, as described for the main experiment. There were 3 repetitions for each of the predetermined distances of 2, 5, and 10 m (9 trials in total), and the order of the distances was randomized. For each trial, sighted participants and blind participants with light perception only were asked to close their eyes and walk forwards over the predetermined distance. No feedback was given.

**Fig. 4:**
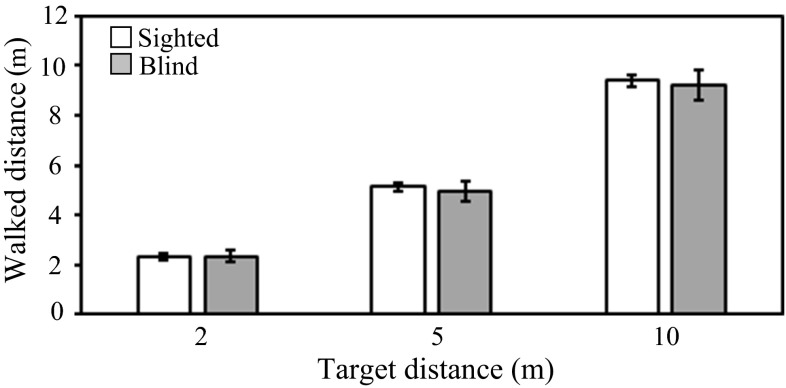
Geometric mean walked distances for sighted participants (*open bars*) and blind participants (*gray bars*), plotted against the target distance. *Error bars* represent ±1 standard error

Figure 4 shows geometric mean walked distances for sighted and blind participants. Walked distances were similar for sighted and blind participants for all three target distances. The mean unsigned error was assessed using a mixed-model ANOVA with distance as a within-subjects factor and blindness as a between-subjects factor. The main effect of distance was significant [*F*(2, 32) = 4.35, *p* < 0.05], but there was no main effect of blindness or interaction (both *p* > 0.05), suggesting that the differences across groups observed in the main experiment were not due to differences in general ability to judge distance between sighted and blind participants.

## Discussion

Blind participants consistently overestimated the distance to nearby simulated sound sources and underestimated the distance to remote sound sources. They did the latter to a greater extent than sighted participants. These findings suggest that without visual information to aid in calibration, the internal representation of virtual auditory distance is compressed for both anechoic and reverberant environments, and across a range of stimuli, consistent with the perceptual deficiency hypothesis.

The judged distance data for sighted participants (Fig. 2) were consistent with effects described in the literature for both real and virtual sound sources, suggesting that the virtual techniques used here offered an acceptable simulation of auditory distance cues. Normally sighted participants consistently underestimated the distance to more distant sound sources, as reported for many studies of real and virtual apparent distance [see Kolarik et al. (2016a) and Zahorik et al. (2005) for reviews]. Judged distances tended to be more veridical for conversational-level speech stimuli (Gardner 1969) than for noise stimuli (Zahorik 2002a), consistent with the view that sighted participants can use their familiarity with the characteristics of speech to improve their judgments of apparent distance (Brungart and Scott 2001). However, it is possible that the longer duration of the speech stimuli contributed to the advantage observed here for those stimuli (the stimuli were chosen to match those used in previous studies and thus differed in duration). Although the simulation utilized nonindividualized HRTFs, it is unlikely that this contributed to the differences observed between blind and sighted participants, as pinna cues do not provide distance information for simulated stimuli placed directly ahead of the participant at distances greater than 1 m (Otani et al. 2009). It is therefore likely that the results of this study give a fair indication of distance judgments made by blind participants in real rooms. However, such judgments have yet to be obtained, and we confine discussion of the current findings to virtual environments only.

Given previous work showing that judgments of sound source azimuth by blind participants are as good as or better than those made by sighted participants, it appears that blind participants hear the direction of sound sources well, but make greater random and systematic errors than sighted participants in judging their distance. In the current study, lower precision was found for the closer sound sources for blind participants than for sighted controls. These results are in agreement with a study of relative auditory distance judgments by Cappagli et al. (2015), who found lower precision for blind than sighted controls for distances outside peripersonal space. The strong compression of auditory space for blind participants might be explained by a limited ability to calibrate distance using audiomotor feedback. Blind participants rarely, if ever, have direct information about the distance of sound sources in extrapersonal space in order to calibrate level and D/R cues. For sighted participants, visual range information about the whole scene increases the accuracy of auditory distance judgments even when the sound source itself is not visible (Zahorik 2001; Calcagno et al. 2012). As only limited range information is available to blind participants using audiomotor feedback, this may prevent the development of accurate internal representations of auditory distance. The current findings are consistent with previous work suggesting that visual information is needed to calibrate auditory space sufficiently accurately to allow good performance in more challenging auditory spatial tasks. For example, Gori et al. (2014) showed that although blind participants were well able to detect a change in azimuth, they displayed deficits when performing an auditory spatial bisection task requiring a representation of space that stayed in memory for a relatively long duration and necessitated the use of a well-calibrated internal topographical spatial map.

It is possible that the point at which blind participants perceive auditory distance without bias correlates with information that they use to estimate distance in everyday life. For example, they may perceive that distance correctly because it correlates with the distance at which they tap with the cane in the floor. Although this was not investigated in the current study as some of our participants did not use a cane (instead relying on a guide dog), further investigation would help to establish whether there is a correlation between point of correct perceived auditory distance for each participant and the distance at which they are used to tapping or generating sound with the cane on the floor, that could suggest that auditory perception can be calibrated by audiomotor feedback.

The compression of internal representations of distance following visual loss may have consequences for navigation. According to representation or model-based control approaches (Frenz and Lappe 2005; Turano et al. 2005), sensory information allows the formation of internal representations of the environment for navigation, and under visual guidance, surrounding space is generally accurately represented in relation to participants’ action capabilities. However, internal spatial representations based on sound are likely coarser than for vision (Milne et al. 2014; Kolarik et al. 2016b). Studies have shown that blind individuals develop and primarily rely on egocentric spatial representations where the body is used as a means to center organization of the surrounding space (Corazzini et al. 2010; Iachini et al. 2014; Schinazi et al. 2016), and egocentric spatial representations could be used as a basis for navigation if the spatial location of a single sound source was used as a goal. However, a relatively coarse compressed auditory internal representation of distance could result in the perceived location of the goal being inaccurate or imprecise during initial path planning. This could affect locomotion by those with sight loss during wayfinding, leading to slower or less accurate movements, as the central nervous system (CNS) would have to compensate for the compression of perceived auditory space to reach the goal.

For farther sound sources, it is possible that the compression of perceived auditory distance associated with blindness provides an evolutionary advantage. Underestimation of the distance of farther sound sources, as occurs for sighted individuals, has been proposed to provide an additional “margin of safety” for navigating safely through the environment and avoiding obstacles using sound (Zahorik et al. 2005), similar to that resulting from the systematic underestimation of the time-to-arrival of approaching sound sources by sighted individuals (Ghazanfar et al. 2002). One possibility is that the greater-than-normal compression of perceived auditory distance for blind individuals reflects an adaptive bias, providing a selective advantage in preparing for contact with a farther sound source and in guiding locomotion. For example, perceiving a farther sound source to be closer than it actually is would promote an earlier response, which would be advantageous if the signal was threatening.

It is not currently known whether relative and absolute distance cues are processed by different neuronal mechanisms. Also, while many studies have investigated the cross-modal reorganization of visual brain regions that may underlie enhanced sound source localization by blind participants (reviewed by Collignon et al. 2009, and Voss et al. 2010), we are not aware of any studies that have investigated whether visual areas are recruited to process auditory distance following sight loss. Further work is needed to link the behavioral evidence for compressive distance judgments presented here and previously (Kolarik et al. 2013c) with neuronal mechanisms of auditory distance perception by blind participants.

The age of onset and duration of sight loss can affect auditory abilities (Voss et al. 2004; Wan et al. 2010) and can affect the extent of cross-modal recruitment in dorsal brain regions in response to auditory spatial information (Dormal et al. 2012). Age of onset of sight loss may also be a factor in the topographical representation of auditory space. For normally sighted listeners, it is possible that the representation of the auditory world is being constantly updated using calibration information from visual signals. When vision is lost, the representation of distance information based on hearing is not maintained and becomes more compressed. If this is so, then a compressed representation of auditory distance should be found even among late-onset blind listeners. Alternatively, the cross-modal calibration hypothesis (Gori et al. 2010) proposes that during development, information from a more accurate sense, such as vision, is used to calibrate another less accurate sense, such as hearing, and that once established the calibration does not need to be renewed or repeated. If this is so, the compression of auditory space should be present in early but not in late-blind listeners.

Previous work has shown that for relative distance tasks, blind listeners display better performance than sighted listeners (Ashmead et al. 1998; Voss et al. 2004), in both anechoic and reverberant virtual environments (Kolarik et al. 2013b), although this was not found by Cappagli et al. (2015). Such tasks do not require the participant to report where in space the sounds are perceived to be. Rather, the tasks depend on a comparison between the acoustic characteristics of the two stimuli, differing in physical characteristics such as level or D/R values. Blind participants are better able to distinguish small differences in level or D/R than sighted participants (Kolarik et al. 2013b), and this can explain their better performance in relative distance judgments. However, absolute judgments of distance depend on internal spatial representations of the external world without the benefit of any reference point or comparison stimuli. The results of the present study suggest that these representations are compressed among blind participants.

Taken together, the findings of the current study and those of Kolarik et al. (2013b) show that blind participants are better able than sighted participants to use small differences in acoustic cues to tell which of two sounds is closer, but are worse at reporting how far away those sound sources are. This parallels previous findings in the vertical dimension, showing that blind participants are often better than sighted participants in judging the relative positions of sound sources in elevation (Ashmead et al. 1998), but show deficits for absolute judgments of elevation (Lewald 2002b; Voss et al. 2015). The current results suggest that any auditory sensory enhancement that develops following severe visual loss does not also result in enhanced accuracy in judging the distance of sounds. Rather, blind people experience a compressed representation of auditory distance, at least when listening to a simulation of single sound sources in quiet anechoic or reverberant rooms. However, further work is needed to investigate the effect of visual loss on absolute distance perception in other situations that occur often in everyday life, such as when background noise and multiple sound sources are present. Although blind individuals are likely to constantly update their spatial representations via multiple sound sources when present, if this occurs within a representation of auditory distance that is compressed overall, accuracy may still be compromised compared to sighted listeners as the absolute distances of the reference sounds may be systematically underestimated in far space.

## Footnotes

1 A control experiment was conducted to assess whether performance differed between conditions where participants wore a blindfold and where they were instructed to keep their eyes closed. Normally sighted participants with normal hearing as described for the main experiment (*n* = 10, 7 males and 3 females, mean age 33 years, range 28–42 years) were tested using reverberant speech only, as it was assumed that any effects of visual information would similarly affect auditory distance judgments for other stimuli. Half of the participants were blindfolded prior to entering the testing room. They then left the room and completed a distractor task (a questionnaire regarding general hearing abilities) for 15 min. They then returned and were tested under instruction to keep their eyes closed, as for the main experiment. The other half of the participants completed the experiment in the reverse order. Similar performance was observed for the eyes closed and blindfolded conditions, and errors increased as virtual distance increased. Errors were evaluated using a within-subjects ANOVA with distance and vision occlusion method as factors. A main effect of distance only was found [*F*(7, 63) = 16.67, *p* < 0.001]. Other main effects and interactions were not significant (all *p* > 0.05), suggesting that participants followed the instructions to keep their eyes closed, and thus visual information did not affect performance.
